# Mechanisms by which environmental regulation and social network embeddedness influence farmers’ ecological efficiency

**DOI:** 10.1038/s41598-025-19184-3

**Published:** 2025-10-08

**Authors:** Li Runze, Zhu Jian, Chen Ke, Wang Yang

**Affiliations:** College of Economics and Management, Shenyang Agricultural University, Shenyang, 110866 China

**Keywords:** Forest farmers, Ecological efficiency, Social network embeddedness, Environmental regulation, Ecology, Ecology, Environmental sciences, Environmental social sciences

## Abstract

Identifying the key drivers of ecological efficiency improvement among forest farmers is essential for advancing the reform of the collective forest tenure system and promoting the modernization of forestry. Based on survey data from 324 hazelnut farmers in Tieling City, Liaoning Province, this study developed an analytical framework that links external regulation, internal network embeddedness, and ecological efficiency. A super-efficiency Slack-Based Measure (SBM) model was employed to assess production efficiency, and the effects of environmental regulation and social network embeddedness on ecological efficiency were systematically investigated, along with their interaction mechanisms. The results showed that environmental regulation had a significant positive effect on ecological efficiency, with coercive regulation exerting the strongest influence, significantly exceeding that of incentive-based and guidance-based approaches. Social network embeddedness also significantly enhanced ecological efficiency, with degree centrality having the most significant impact, followed by betweenness and closeness centrality. Moreover, the interaction effects between environmental regulation and centrality indicators displayed heterogeneous characteristics. Based on these findings, the study proposes three policy implications: (1) the government should strengthen regulatory enforcement and fiscal incentives, complemented by awareness campaigns and technical training to improve farmers’ environmental consciousness; (2) farmer cooperation and information-sharing platforms should be encouraged to enhance capacity building and optimize social network structures; (3) differentiated policy interventions should be developed according to interaction patterns to leverage network-driven spillovers, motivating farmers to act as role models in the green transition and improving regulatory effectiveness.

## Introduction

Globally, agricultural production faces the dual challenge of increasing output and ensuring food security while maintaining ecological protection and sustainable resource use. Evidence shows that higher productivity is often accompanied by greater environmental pressure^1^. Farm diversification, while improving ecological performance, can generate tensions with managerial efficiency and cost control^2^. Agricultural specialization brings considerable economic returns but results in deforestation and biodiversity loss^3^. These findings highlight that the tension between economic development and ecological protection is a universal feature of agricultural systems. Against this global backdrop, economic forests, as a vital component of China’s forestry economy, deliver significant economic, social, and ecological benefits. Their performance directly influences rural development, income growth among forest-dependent households, and ecological conservation within collective forest regions^4^. In 2008, a new phase of collective forest tenure reform was initiated nationwide, extending the household contract responsibility system to forestlands. The reform led to structural optimization within the forestry sector, improved the efficiency of forest tenure transfer and resource allocation, and stimulated productivity gains in economic forest management^5–7^. By 2023, the total area under economic forest cultivation had reached 466,669 square kilometers, involved nearly 90 million rural laborers and produced an output value exceeding 2 trillion yuan. Economic forests have become an essential foundation for advancing the holistic food system and securing income growth for households in collective forest zones^8^. Despite the notable increase in productivity driven by the reform, challenges persist in achieving sustainable resource utilization and ecological protection. Several key constraints continue to limit improvements in ecological efficiency^9,10^. Weak ecological awareness has hindered the alignment of production practices with environmental objectives, leading to ongoing ecological stress. The pursuit of short-term economic gains and inappropriate application of chemical inputs have contributed to soil degradation and water pollution. In addition, extensive and inefficient resource management has further constrained sustainable development^11–14^. As the ongoing deepening of collective forest tenure reform and efforts to modernize forestry continue, enhancing the ecological efficiency of economic forests has emerged as a critical issue demanding urgent attention.

Existing studies have identified environmental regulation as a crucial external force driving the adoption of green production practices among forest farmers. Through mandatory compliance, economic incentives, and behavioral guidance, environmental regulation restructures external constraint mechanisms, suppresses opportunistic behavior, and fosters the adoption of environmentally sustainable production methods^15–19^. Further evidence also suggests that regulatory effects are not “one-size-fits-all”; farmers’ ecological responses vary significantly under different instruments and levels of regulatory intensity, with potential nonlinear characteristics^20,21^.At the same time, social network embeddedness operates as a vital endogenous mechanism that promotes the transformation toward green production by improving access to information, accelerating the diffusion of technology, and facilitating resource integration^22–24^. First, embedded networks enhance information acquisition through interpersonal relationships and market intermediaries, increasing farmers’responsiveness to green technologies, market dynamics, and policy signals, thereby improving production decision-making^25,26^. Second, farmers with stronger network embeddedness are more likely to access mutual assistance and technical resources, leading to higher efficiency in resource allocation^27^. Third, social network embeddedness strengthens farmers’ capacity for collective action, enabling cooperation with associations, family forest farms, and leading enterprises. Such collaboration supports resource sharing, risk diversification, and cost reduction, creating favorable conditions for the adoption of green production practices^28,18^.

Although prior studies provide valuable theoretical foundations for the current research, several important gaps remain. First, existing literature on ecological efficiency among forest farmers lacks in-depth investigation. Most existing studies focus on the willingness and behavior regarding green technology adoption^29,30^, while systematic evaluations of the effectiveness of green practices remain limited. Second, the measurement of social network embeddedness often suffers from subjectivity, as it typically relies on self-reported questionnaires^31–33^, which may fail to accurately capture farmers’ actual positions and structural influence within networks. Objective and systematic quantitative approaches are still lacking. Third, research on the interaction between internal and external factors affecting ecological efficiency remains weak. While previous research has considered individual influences such as external constraints and personal endowments^27,34^, few studies have established an integrated theoretical framework capable of revealing the synergistic pathways through which these variables interact. The present study makes three principal marginal contributions. First, a super-efficiency slack-based measure (SBM) model is employed to construct an ecological efficiency evaluation framework, enabling a comprehensive assessment of multidimensional inputs and outputs and enhancing the scientific rigor and explanatory power of efficiency measurement. Second, social network analysis methods are introduced, employing structural indicators such as degree centrality, betweenness centrality, and closeness centrality to objectively quantify the extent of social network embeddedness among forest farmers, addressing the limitations of subjective measurement approaches in prior studies. Third, an interaction effect model incorporating both environmental regulation and social network embeddedness is constructed to uncover the joint mechanism through which these factors influence ecological efficiency, thereby providing empirical evidence for policy guidance on green behavioral transformation. Through the above research, this study not only responds to the global challenge of reconciling agricultural productivity growth with ecological protection, revealing the general patterns of improving ecological efficiency under the framework of sustainable development; but also deepens the understanding of green transformation pathways for farmers in the specific context of China’s economic forests; and ultimately clarifies the interaction mechanisms between environmental regulation and social network embeddedness, providing empirical support for enhancing ecological efficiency and optimizing policy design in economic forest management.

## Theoretical analysis and research hypotheses

Drawing on the theory of interactionism^35^, the present study develops an analytical framework that integrates external regulation, internal embeddedness, and ecological efficiency. The framework aims to systematically examine how environmental regulation and social network embeddedness jointly influence the improvement path of ecological efficiency among forest farmers.

### Mechanisms through which environmental regulation influences ecological efficiency among forest farmers

The improvement of ecological efficiency is often constrained by typical negative externalities, which necessitate effective policy interventions. According to the Environmental Kuznets Curve (EKC) hypothesis, ecological stress and income levels follow an inverted U-shaped relationship^36^. In the early stages of economic development, forest farmers tend to neglect ecological protection, resulting in rising environmental risks. To address these externalities, Pigouvian theory and Coase’s theorem propose two distinct approaches. The former advocates for government intervention through taxes or subsidies to internalize external costs^37^, while the latter emphasizes clarifying property rights and utilizing market mechanisms to achieve efficient outcomes^38^. However, in rural contexts, unclear ecological property rights and underdeveloped trading mechanisms significantly limit the applicability of the Coasean approach. Consequently, government-led environmental regulation, grounded in Pigouvian logic, has emerged as a critical institutional tool for enhancing ecological efficiency in forest regions. As a key form of external institutional intervention, environmental regulation is typically classified into three categories: coercive, incentive-based, and guidance-oriented regulation^39–41^. Coercive regulation imposes rigid standards and punitive mechanisms that create external pressure, compelling forest farmers to comply with green norms and effectively curbing resource waste and pollution^42^. Incentive-based regulation reduces the marginal cost of green transformation through subsidies, rewards, and technical training, thereby enhancing sustainable development capacity^15^. Guidance-oriented regulation relies on non-coercive dissemination of environmental information and knowledge, stimulating farmers’ environmental awareness and voluntary green practices and subtly contributing to ecological efficiency improvements^43^. Based on this theoretical foundation, the following hypotheses are proposed:

#### H1

Environmental regulation exerts a significant positive effect on the ecological efficiency of forest farmers.

#### H1a

Coercive regulation exerts a significant positive effect on the ecological efficiency of forest farmers.

#### H1b

Incentive-based regulation exerts a significant positive effect on the ecological efficiency of forest farmers.

#### H1c

Guidance-oriented regulation exerts a significant positive effect on the ecological efficiency of forest farmers.

### Mechanisms through which social network embeddedness influences ecological efficiency among forest farmers

According to social capital theory, resource sharing, information exchange, and cooperative mechanisms embedded within social networks can significantly improve the efficiency of individual behavior^44^. Building on previous research, social network embeddedness can be measured using three structural centrality indicators: degree centrality, betweenness centrality, and closeness centrality^45,46^. Degree centrality refers to the number of direct connections a node maintains, representing the breadth of information inflow and the scope of social coverage^47^. Forest farmers with a higher degree of centrality typically enjoy more robust access to information, allowing for timely updates on green technologies, policy developments, and market trends. This access contributes to more informed and efficient ecological decision-making. Betweenness centrality reflects the extent to which a node occupies a bridging position within network pathways. Farmers with high betweenness centrality often act as critical intermediaries in the flow of information, facilitating the dissemination of green technologies and environmental policy resources across otherwise disconnected groups. This intermediary role promotes broader diffusion of sustainable practices and contributes to enhanced ecological efficiency^48^. Closeness centrality captures the average shortest distance between a node and all other nodes in the network, indicating the efficiency with which information and resources can be accessed^49^. Farmers with high closeness centrality are typically located near the network core, enabling rapid and comprehensive access to information. This positional advantage enhances the timeliness and precision of ecological decision-making, supporting the optimization of green behaviors and resource allocation strategies. Based on the above theoretical rationale, the following hypotheses are proposed:

#### H2

Social network embeddedness has a significant positive effect on the ecological efficiency of forest farmers.

#### H2a

Degree centrality has a significant positive effect on the ecological efficiency of forest farmers.

#### H2b

Betweenness centrality has a significant positive effect on the ecological efficiency of forest farmers.

#### H2c

Closeness centrality has a significant positive effect on the ecological efficiency of forest farmers.

### Synergistic effects of environmental regulation and social network embeddedness

In the theoretical framework of this study, the ecological efficiency of forest farmers ($\:\mu\:$) is jointly influenced by environmental regulation ($\:\text{E}\text{R}$) and social network embeddedness ($\:SNE$), representing the interplay between external institutional forces and internal social capital. These two dimensions exert a synergistic effect in promoting ecological efficiency, while farmers, guided by the principle of utility maximization, make decisions aimed at improving ecological outcomes. Accordingly, the ecological efficiency of forest farmers ($\:\mu\:$)can be modeled as follows:

$$\:\begin{array}{c}\mu\:=\mu\:\left(ER,SNE\right)\#（1）\end{array}$$

Where $\:ER$ and $\:SNE$ represent environmental regulation and social network embeddedness, respectively. Given the potential joint influence of environmental regulation and social network embeddedness on ecological efficiency, an extended specification is constructed to capture their synergistic (moderating) effect. Accordingly, the following synergy-oriented specification is proposed:

$$\:\begin{array}{c}SNE=S\left(ER\right)\:\#（2）\end{array}$$

$$\:\begin{array}{c}\:EL=ER\times\:f\left(SNE\right)\#（3）\end{array}$$

To further analyze the utility function $\:\mu\:$, a differential approach is applied under the following assumptions: First, both $\:ER$ and $\:SNE$ are positive and persist over time. Environmental regulation remains stable at the policy level, while social network embeddedness is closely tied to farmers’ decision-making behaviors. Second, the utility effects of both factors increase over time. Environmental regulation requires long-term enforcement to yield results, whereas social network embeddedness gradually exerts its influence through the accumulation of information and resources. Third, the marginal effects of both environmental regulation and social network embeddedness on ecological efficiency exhibit diminishing returns. As ecological efficiency improves, the incremental contribution of each factor decreases accordingly.

$$\:\begin{array}{c}\frac{\partial\:\mu\:}{\partial\:ER}>0,\frac{{\partial\:}^{2}\mu\:}{\partial\:E{R}^{2}}<0;\frac{\partial\:\mu\:}{\partial\:SNE}>0,\frac{{\partial\:}^{2}\mu\:}{\partial\:SN{E}^{2}}<0\#（4）\end{array}$$

Under the stated assumptions, the utility function $\:\mu\:$ is further differentiated. Through the application of differential calculus and variable substitution, the following utility function is derived and expressed in a simplified form:

$$\:\begin{array}{c}d\mu\:={\xi\:}_{ER}\times\:\frac{dER}{ER}+{\xi\:}_{SNE}\times\:\frac{dSNE}{SNE}\#（5）\end{array}$$

$$\:\begin{array}{c}d\mu\:={\xi\:}_{ER}\times\:\frac{dER}{ER}+{\xi\:}_{SNE}\times\:\frac{dSNE}{SNE}\left[1+{\xi\:}_{ER}\times\:\left(\frac{\partial\:ER}{\partial\:SNE}\right)\right]\#（6）\end{array}$$

Based on the utility maximization hypothesis, the analytical framework further suggests that the ecological efficiency of forest farmers is not only directly influenced by environmental regulation but also significantly shaped by its interaction with social network embeddedness. Accordingly, the following hypothesis is proposed:

#### H3

The interaction between environmental regulation and social network embeddedness has a significant positive effect on the ecological efficiency of forest farmers.

**Fig. 1:**
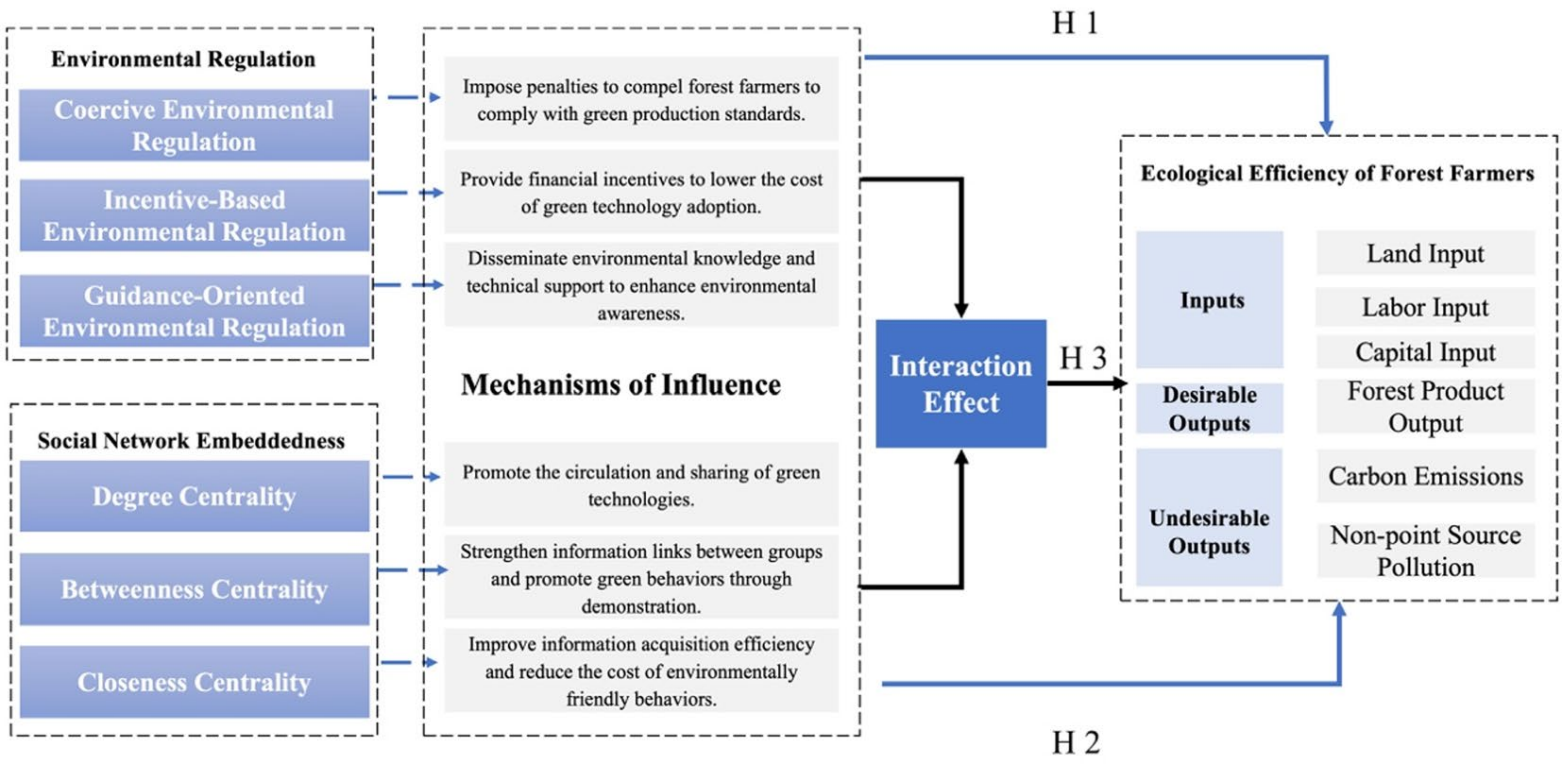
Theoretical framework.

Building on the preceding analysis, this study adopts social network embeddedness and environmental regulation as the core analytical dimensions to construct a multidimensional theoretical framework. The framework systematically explores the internal and external mechanisms driving improvements in ecological efficiency among forest farmers, as illustrated in Fig. 1, and the joint effect proposed here will be empirically tested through interaction terms in the subsequent analysis (Eq. 20).

## Data source and variable description

### Data source

The data for this study were obtained from a field survey conducted in January 2024 by the research team among hazelnut (*Corylus heterophylla Fisch*) growers in Tieling City, Liaoning Province. As one of China’s major hazelnut production areas, Tieling reported a total hazelforest area of 753.34 square kilometers and an annual output of 31,000 tons in 2022, ranking first nationwide in both cultivation area and yield^50^. The city is home to approximately 18,000 hazelnut-farming households, and the industry has become a key driver of rural income growth^51^. Despite this economic importance, some farmers have prioritized short-term returns over sustainable resource use, resulting in frequent ecological issues such as soil degradation and water pollution. To examine the mechanisms underlying ecological efficiency improvement in economic forest management and to inform the ongoing reform of the collective forest tenure system, the survey was conducted within the hazelnut Geographical Indication (GI) protection zone in Tieling. A stratified random sampling approach was employed. Tieling County awarded the title of “China’s Leading Hazelnut Industry County,” and Kaiyuan City, recognized as a “National Demonstration County for the Hazelnut Industry,” were selected as sample areas to ensure typicality and representativeness. In each county, five villages were randomly chosen, yielding a total of ten sample villages. Within each village, 25 to 30 households were randomly surveyed. A total of 332 questionnaires were distributed, and after excluding invalid responses, 324 valid questionnaires were obtained, resulting in an effective response rate of 97.5%.

In compliance with ethical standards, the survey protocol was reviewed and approved by the College of Economics and Management, Shenyang Agricultural University. All procedures were carried out in accordance with institutional guidelines and relevant national regulations. Before data collection, all participants signed written informed consent forms after being clearly informed of the purpose of the study, the voluntary nature of their participation, their right to withdraw at any time, and the confidentiality of their responses.

### Variable selection and descriptive statistics

#### Dependent variable

The dependent variable in this study is ecological efficiency, as it comprehensively reflects the balance between resource utilization, output generation, and environmental impact, and is widely recognized as a key indicator for measuring the sustainability of green production. Following the methodology adopted in prior research^52,53^, five input variables were selected: land area under hazelnut cultivation, fertilizer expenditure, pesticide expenditure, hired labor costs, and family labor time. The expected output is defined as total household income from hazelnut production, while undesirable outputs include carbon emissions and non-point source pollution, allowing for a comprehensive assessment of efficiency.

**(1) Carbon emissions calculation**.

Carbon emissions in hazelnut production primarily stem from three types of inputs: First, emissions associated with the production, transportation, and application of fertilizers, including nitrogen, phosphorus, potassium, and compound fertilizers. Second, indirect emissions are generated during pesticide use. Third, direct emissions resulting from diesel-powered machinery used in field management. Emission levels were calculated using the actual quantities of inputs reported by each household, combined with standard carbon emission coefficients. The emission factors were primarily based on estimates from the Oak Ridge National Laboratory (ORNL) in the United States and were further adjusted using the correction parameters proposed by Dubey et al. Detailed coefficients and calculation parameters are presented in Table 1.

**Table 1:** Carbon emission factors for input sources.

Indicator	Input Type		Emission Coefficient	Source
Carbon emissions (input consumption /unit area×emission coefficient	Fertilizer	Phosphorus fertilizer	0.66 kg C/kg	CLCD 0.7 ^54^
		Nitrogen fertilizer	2.39 kg C/kg	
		Potassium fertilizer	0.57 kg C/kg	
		Compound fertilizer	1.77 kg C/kg	
	Pesticide		4.934 kg C/kg	Oak Ridge National Laboratory ^55^
	Diesel		0.592 kg C/km^2^	IPCC ^56^

**(2) Measurement of non-point source pollution**.

Fertilizer application is one of the primary contributors to non-point source pollution. The levels of total phosphorus (TP) and total nitrogen (TN) per unit of the cultivated area were used as proxies for pollution intensity. The calculation formulas are as follows:

$$\:\begin{array}{c}TP=\left({P}_{u}\times\:{P}_{c}\times\:{P}_{l}\right)+\left(CM{P}_{u}\times\:{P}_{c}\times\:{P}_{l}\right)\#（7）\end{array}$$

$$\:\begin{array}{c}TN=\left({N}_{u}\times\:{N}_{c}\times\:{N}_{l}\right)+\left({K}_{u}\times\:{N}_{c}\times\:{N}_{l}\right)\#（8）\end{array}$$

Where $\:{P}_{u}$ is the amount of phosphate fertilizer applied, $\:{P}_{c}$is the phosphorus content coefficient in phosphate fertilizer, $\:{P}_{l}$ represents the phosphorus loss rate, $\:CM{P}_{u}$ is the amount of compound fertilizer applied, $\:{N}_{u}$ is the amount of nitrogen fertilizer applied, $\:{N}_{c}$ is the nitrogen content coefficient in nitrogen fertilizer, $\:{N}_{l}$ is the nitrogen loss rate, $\:{K}_{u}$ is the amount of potassium fertilizer applied. The pollution production coefficients and runoff loss rates for each fertilizer type were based on the empirical results of Lai (2004). Accordingly, the TP and TN emissions were calculated using the following equations:

$$\:\begin{array}{c}TP={P}_{u}\times\:0.44\times\:4\%+CM{P}_{u}\times\:0.15\times\:4\%\#（9）\end{array}$$

$$\:\begin{array}{c}TN={N}_{u}\times\:1\times\:20\%+CM{P}_{u}\times\:0.33\times\:20\%\#（10）\end{array}$$

**(3) Measurement of ecological efficiency among forest farmers**

By integrating key input variables such as forestland area, fertilizer costs, pesticide costs, and labor input, along with the expected output of forestry production income and the undesirable outputs of carbon emissions and non-point source pollution, ecological efficiency among forest farmers is evaluated using the super-efficiency Slack-Based Measure (SBM) model. Slack variables are introduced to capture the discrepancy between inputs and outputs, allowing for a more precise identification of efficiency variations. The model is specified as follows:

$$\:\begin{array}{c}{\:\theta\:}^{*}=min\left(\frac{1+\frac{1}{m}\sum\:_{i=1}^{m}\frac{{s}_{i}}{{x}_{i}}}{1-\frac{1}{q+h}\left(\sum\:_{r=1}^{q}\frac{{s}_{r}^{+}}{{y}_{r}}+\sum\:_{k=1}^{h}\frac{{s}_{k}^{-}}{{b}_{k}}\right)}\right)\#（11）\end{array}$$subject to:

$$\:\begin{array}{c}{x}_{io}\ge\:\sum\:_{t=1}^{T}\sum\:_{N=1}^{N}{\lambda\:}_{j}{x}_{ij}-{s}_{i},i=\text{1,2},\dots\:,m\#（12）\end{array}$$

$$\:\begin{array}{c}{y}_{to}\ge\:\sum\:_{t=1}^{T}\sum\:_{N=1}^{N}{\lambda\:}_{j}{y}_{rj}+{s}_{r}^{+},r=\text{1,2},\dots\:,q\#（13）\end{array}$$

$$\:\begin{array}{c}{b}_{ko}\ge\:\sum\:_{t=1}^{T}\sum\:_{N=1}^{N}{\lambda\:}_{j}{b}_{kj}-{s}_{k}^{-},k=\text{1,2},\dots\:,h\#（14）\end{array}$$

$$\:\begin{array}{c}{s}_{i}\ge\:0,{s}_{r}^{+}\ge\:0,{s}_{k}^{-}\ge\:0,\forall\:i,\forall\:r,\forall\:k\#（15）\end{array}$$

Let $\:{x}_{i}$ denote the *i*-th input variable, including forest land area, fertilizer costs, pesticide costs, hired labor costs, and family labor time. Let $\:{y}_{r}$ represent the *r*-th desirable output variable, specifically the household income derived from forest production. Let $\:{b}_{k\:}$denote the *k*-th undesirable output variable, including carbon emissions and non-point source pollution. The term $\:{s}_{i}$ represents the slack variable associated with the input *i*, indicating inefficiencies in resource use. The term $\:{s}_{r}^{+}$ denotes the slack in the desirable output $\:{y}_{r}$, while $\:{s}_{k}^{-}$ corresponds to the slack in the undesirable output $\:{b}_{k}$. Descriptive statistics for the input, desirable output, and undesirable output variables used to estimate ecological efficiency among forest farmers are presented in Table 2.

**Table 2:** Descriptive statistics of input and output variables and ecological efficiency among forest farmers.

Variable	Definition and Description	Mean	Std. Dev.	Min	Max
Forest land area	Actual area used by farmers for forest product cultivation (km²)	0.0324	0.0357	0.00133	0.3333
Fertilizer cost	Total expenditure on fertilizers (CNY)	3593.333	5836.062	0	80,000
Pesticide cost	Total expenditure on pesticides (CNY)	3386.136	6042.309	0	90,000
Hired labor cost	Total cost of hiring external labor (CNY)	8016.420	17455.520	0	200,000
Family labor hours	Labor hours contributed by family members to forest production (hours)	1511.988	923.329	242	5400
Forestry income	Income generated from the sale of forest products (CNY)	48485.280	51669.700	1800	500,000
Carbon emissions	Carbon emissions produced during the forestry production process (kg C)	2483.383	2425.018	101	30,409
Non-point source pollution	Non-point source pollution generated during production (TP + TN)	77.908	93.519	0	1272.187
Ecological efficiency	Ecological efficiency score calculated using the SBM (Slack-Based Measure) model	0.121	0.184	0.005	1

#### Independent variables

**(1) Environmental regulation**

Because environmental regulation serves as a key external institutional force that significantly shapes farmers’ production behavior, this study, drawing on existing literature^41^ and based on the distinct mechanisms through which policy interventions operate, environmental regulation in this study is categorized into three dimensions: coercive regulation, incentive-based regulation, and guidance-based regulation. The specific survey items used to measure each dimension are presented in Table 3. In this study, all items in Table 3 were evaluated using a five-point Likert scale (1 = “strongly disagree " to 5 = “strongly agree “). For each regulatory dimension—coercive, incentive-based, and guidance-oriented—the mean value of the corresponding items was calculated. The composite environmental regulation index was then derived by aggregating the three-dimension scores, resulting in a continuous measure of overall regulatory intensity.

**Table 3:** Survey items and descriptive statistics for environmental regulation variables.

Variable Type	Survey Item	Mean	Std. Dev.
Coercive Regulation	If high-pollution pesticides banned by the government are used, will the responsible authority penalize or report you?	0.836	0.370
	If the amount of pesticide used exceeds the government’s specified limits, will this result in disqualification from GI certification?	0.407	0.492
	Does the government regularly inspect or supervise the use of pesticides and fertilizers during production?	0.632	0.482
Incentive-Based Regulation	Does the government provide subsidies for using organic fertilizers and biopesticides?	0.496	0.501
	Can green production practices allow your agricultural products to receive price premiums in the market?	0.290	0.454
	Does adopting organic fertilizers and biopesticides improve your personal reputation or recognition within the community?	0.817	0.386
Guidance-Based Regulation	Have you ever participated in technical training on organic fertilizers or biopesticides organized by the government or cooperatives?	0.280	0.450
	Have you reduced the use of chemical fertilizers or conventional pesticides due to the green production practices demonstrated by other farmers in your village?	0.503	0.500
	Have government or cooperative promotions influenced your belief that organic fertilizers and biopesticides yield higher long-term benefits?	0.697	0.460

**(2)Social network embeddedness**

Social network embeddedness is regarded as an important endogenous factor that influences farmers’ access to information, coordination of resources, and participation in collective actions, thereby exerting a significant impact on ecological efficiency. In the existing literature, it is typically measured through centrality indicators, which effectively capture farmers’ position and connectivity within social networks^36,38^. Building on this, the present study characterizes social network embeddedness from three dimensions—connection breadth, bridging capacity, and efficiency of information transmission—by employing degree centrality, betweenness centrality, and closeness centrality. Principal Component Analysis (PCA) was then applied to integrate these indicators into a composite index representing overall network embeddedness. The PCA results show that the KMO statistic is 0.676, and Bartlett’s test yields X² = 317.98 (*p* < 0.001), indicating that the data are suitable for factor analysis. The eigenvalue of the first principal component is 2.138, which explains 71.04% of the total variance, suggesting that the constructed composite index effectively reflects the common structure of social network embeddedness.

Degree centrality quantifies the number of direct connections a node holds, indicating the level of activity within the network and the breadth of access to resources. The formula for calculating degree centrality is as follows:

$$\:\begin{array}{c}{C}_{{D}_{i}}=\frac{{d}_{i}}{N-1}\#（16）\end{array}$$

Let $\:{\text{C}}_{{\text{D}}_{\text{i}}}$ denote the degree centrality of node$\:\:i,\:\:{\text{d}}_{i}\:$represent the number of direct connections of node $\:i$, and N be the total number of nodes in the network.

Betweenness centrality captures the extent to which a node serves as a bridge along the shortest paths connecting other nodes, reflecting its importance in the flow of information and coordination of resources. The corresponding calculation formula is as follows:

$$\:\begin{array}{c}{C}_{{B}_{i}}=\sum\:_{s\ne\:i\ne\:t}\frac{{\sigma\:}_{st}\left(i\right)}{{\sigma\:}_{st}}\#（17）\end{array}$$

Where $\:{{\upsigma\:}}_{\text{s}\text{t}}$ denotes the total number of shortest paths from node $\:s$ to node $\:t$ and $\:{{\upsigma\:}}_{\text{s}\text{t}}\left(\text{i}\right)$ represents the number of those paths that pass-through node $\:i$. The betweenness centrality formula measures the proportion of shortest paths that include node $\:i$, reflecting its significance in information dissemination.

Closeness centrality measures the average shortest path distance from a given node to all other nodes in the network. This metric evaluates the efficiency with which a node accesses information and resources. The corresponding formula is defined as:

$$\:\begin{array}{c}{C}_{{C}_{i}}=\frac{1}{\sum\:_{j}d\left(i,j\right)}\#（18）\end{array}$$

Where $\:{\text{C}}_{{\text{C}}_{\text{i}}}$ denotes the closeness centrality of node $\:i$, and $\:\text{d}(\text{i},\text{j})$ represents the shortest path length from node $\:i$ to node $\:j$.

#### Control variables

To mitigate estimation bias caused by omitted variables, control variables were selected based on relevant literature and data availability^52,53^. These variables include individual characteristics of forest farmers, household attributes, and regional factors. Detailed definitions and coding schemes are presented in Table 4.

**Table 4:** Variable descriptions.

Variable Name		Definition and Description	Mean	Std. Dev.	Min.	Max.
Dependent	Ecological Efficiency	Ecological efficiency of forest farmers, calculated using the Slack-Based Measure (SBM) model	0.121	0.184	0.005	1
Explanatory	Social Network Embeddedness	Composite index derived from principal component analysis (PCA) of degree centrality, betweenness centrality, and closeness centrality	1.5 × 10^− 16^	1.468	-3.505	8.494
	Degree Centrality	Number of direct connections a household maintains within the social network	0.077	0.041	0.025	0.317
	Betweenness Centrality	The extent to which a household acts as a bridge in the network between other nodes	0.073	0.063	0	0.397
	Closeness Centrality	Average shortest path length from the household to all other nodes in the network	0.33	0.07	0.037	0.578
	Environmental Regulation	Aggregate score of three dimensions of regulation: coercive, incentive-based, and guiding	4.966	2.931	0	9
	Coercive Environmental Regulation	Regulation is enforced through legal penalties to ensure compliance with environmental standards	1.876	1.086	0	3
	Incentive-Based Environmental Regulation	Regulation involving subsidies or rewards to encourage environmentally friendly practices	1.604	1.043	0	3
	Guidance-based Environmental Regulation	Regulation using training, education, and awareness programs to promote environmental consciousness	1.200	0.869	0	3
Control	Age of Hazelnut Farmer	Age of the household head (years)	56.145	8.699	29	64
	Gender of Hazelnut Farmer	Gender of the household head (binary variable)	0.744	0.437	0	1
	Self-Reported Health Status	Health condition self-evaluated by the household head (scaled variable)	3.765	0.735	2	5
	Political Affiliation	Political status of the household head	0.398	0.49	0	1
	Education Level	Highest education level of the household head (scaled variable)	3.359	1.578	0	7
	Years of Hazelnut Cultivation	Number of years the household has been engaged in hazelnut cultivation	12.463	7.746	2	50
	Number of Agricultural Machines	Total number of machines owned for forestry production	1.071	1.109	0	3
	Distance to Nearest Market	Distance between the household’s residence and the nearest agricultural market (km)	1.528	0.683	1	3

## Empirical analysis

### Model specification

To examine the effects of environmental regulation and social network embeddedness on the ecological efficiency of forest farmers, an Ordinary Least Squares (OLS) regression model is employed as the primary analytical framework. A stepwise regression approach is also used to ensure robustness and to identify the most significant explanatory variables. The model is specified as follows:

$$\:\begin{array}{c}{Y}_{i}={\beta\:}_{0}+{\beta\:}_{1}{X}_{i}+{\alpha\:}_{1}{M}_{i}+{\gamma\:}_{1}{Z}_{i}+{\delta\:}_{1}{W}_{i}+{\theta\:}_{1}{Q}_{i}+{ϵ}_{i}\#\left(19\right)\end{array}$$

In Eq. (19)$\:,\:{\text{Y}}_{\text{i}}$ denotes the ecological efficiency of forest farmer $\:\text{i}$, $\:{\text{X}}_{\text{i}}$ represents the explanatory variable for environmental regulation, comprising three components: coercive regulation, incentive-based regulation, and guidance-based regulation. $\:{\text{M}}_{\text{i}}$captures social network embeddedness, measured by degree centrality, betweenness centrality, and closeness centrality. $\:{\text{Z}}_{\text{i}}$ includes basic individual characteristics such as age, political affiliation, education level, gender, and health status. $\:{\text{W}}_{\text{i}}$ refers to production-related variables, including years engaged in hazelnut cultivation and the number of forestry-related machines owned by the household. $\:{\text{Q}}_{\text{i}}$ accounts for regional factors, such as the distance to the nearest market.$\:{\beta\:}_{0},\:{\beta\:}_{1},\:{\gamma\:}_{1},\:{\delta\:}_{1}\:\text{a}\text{n}\text{d}\:{\theta\:}_{1}$ denote the regression coefficients of the corresponding variables, and $\:{\text{ϵ}}_{\text{i}\:}$is the error term.

To further explore the interaction between environmental regulation and social network embeddedness, Eq. (20) introduces an interaction term $\:{\lambda\:}_{i}（{X}_{i}\times\:{M}_{i}）$, following the definition of collaborative efficiency proposed by Wen (2005). This specification is used to test whether the combined effect of institutional regulation and network embeddedness influences ecological efficiency among forest farmers.

$$\:\begin{array}{c}{Y}_{i}={\beta\:}_{0}+{\beta\:}_{1}{X}_{i}+{\alpha\:}_{1}{M}_{i}+{\lambda\:}_{i}（{X}_{i}\times\:{M}_{i}）+{\gamma\:}_{1}{Z}_{i}+{\delta\:}_{1}{W}_{i}+{\theta\:}_{1}{Q}_{i}+{ϵ}_{i}\#\left(20\right)\end{array}$$

### Baseline regression results

A stepwise regression approach was employed to examine the effects of environmental regulation and social network embeddedness on the ecological efficiency of forest farmers. Model (1) assessed the overall impact of environmental regulation, while Models (2) and (3) further explored the distinct effects of the three regulation types. Model (4) incorporated the social network embeddedness variable, and Models (5) and (6) disaggregated the dimensions of embeddedness to evaluate their respective influences on ecological efficiency. The baseline regression results are presented in Table 5. Prior to regression analysis, multicollinearity tests were conducted for all variables. Variance inflation factors (VIFs) calculated using Stata 17.0 were all below 3, indicating low multicollinearity and minimal interference with model estimation.

Regression results indicated a significant positive relationship between environmental regulation and ecological efficiency, with a coefficient of 0.023 significant at the 1% level, supporting Hypothesis H1. Further analysis revealed that coercive regulation exerted the strongest positive effect, with a coefficient of 0.063, exceeding that of incentive-based regulation (0.049) and guidance-based regulation (0.044), thereby validating Hypotheses H1a, H1b, and H1c. Command-based regulation imposed strict compliance through enforcement, thereby encouraging adherence to ecological standards. Incentive-based regulation reduced the cost of green production through subsidies, price premiums, and reputational rewards, strengthening participation willingness. Guidance-based regulation enhanced ecological awareness and technical skills through training and outreach, promoting voluntary adoption of sustainable practices and contributing to systematic improvement in ecological efficiency.

Models (2) to (6) further confirmed the positive effect of social network embeddedness on ecological efficiency, with a regression coefficient of 0.027 significant at the 1% level, supporting Hypothesis H2. Degree centrality consistently exhibited a significant effect across all models, indicating that direct social connections contributed to improved ecological efficiency, confirming Hypothesis H2a. Betweenness centrality also showed a significant positive influence, validating Hypothesis H2b. Forest farmers occupying bridging positions in the network were able to access cross-group resources, including ecological technologies, market opportunities, and policy information, thus facilitating green transformation. Closeness centrality similarly demonstrated a significant positive association. Higher information accessibility enabled forest farmers to respond promptly to policy and technological changes, thereby improving ecological efficiency and confirming Hypothesis H2c.

**Table 5:** Stepwise regression results.

Variable Name	Model (1)	Model (2)	Model (3)	Model (4)	Model (5)	Model (6)
	Forest Farmer Eco-efficiency					
Environmental Regulation	0.023*** (0.003)					
Coercive Regulation		0.061*** (0.009)	0.063*** (0.010)			
Incentive-based Regulation		0.060*** (0.009)	0.049*** (0.013)			
Guidance-based Regulation		0.041*** (0.011)	0.044*** (0.012)			
Social Network Embeddedness				0.027*** (0.007)		
Degree Centrality					1.064*** (0.284)	1.056*** (0.283)
Betweenness Centrality					0.345** (0.196)	0.407** (0.197)
Closeness Centrality					0.021*** (0.162)	0.037*** (0.168)
Farmer Age	0.003*(0.001)		0.003*(0.002)	0.003*(0.001)		0.003*(0.001)
Political Affiliation of the Farmer	0.018** (0.024)		0.010** (0.025)	0.019** (0.025)		0.027** (0.024)
Education Level of the Farmer	-0.022 (0.014)		-0.012 (0.014)	-0.018 (0.024)		-0.027 (0.024)
Gender of the Farmer	0.023 (0.023)		0.028 (0.024)	0.019* (0.024)		0.014 (0.023)
Self-rated Health Status	0.009 (0.013)		0.013 (0.014)	0.012 (0.014)		0.009 (0.013)
Years Engaged in Hazelnut Cultivation	-0.001 (0.001)		-0.001 (0.009)	0.003** (0.015)		0.001** (0.005)
Number of Agricultural Machines Owned	-0.010 (0.009)		− 0.002 (0.009)	-0.003 (0.013)		-0.009 (0.008)
Distance to the Nearest Marketplace (km)	0.001 (0.001)		0.001 (0.001)	0.010* (0.013)		0.001* (0.001)
Constant	-0.164(0.098)	0.024 (0.017)	-0.179*(0.103)	-0.128(0.103)	-0.003 (0.047)	-0.234(0.108)
n	324					
R^2^	0.127	0.117	0.075	0.049	0.136	0.188

Note: *, **, and *** indicate significance at the 10%, 5%, and 1% levels, respectively. The same notation applies hereafter.

### Endogeneity test

In the baseline regressions, the model may suffer from potential reverse causality and omitted variable bias. On the one hand, farmers with higher ecological efficiency are more likely to be included in inspections, training, and publicity programs, thereby perceiving a higher intensity of environmental regulation. On the other hand, improvements in ecological efficiency may encourage farmers to expand information exchange and cooperative participation, thus enhancing their social network embeddedness. To address these endogeneity concerns, this study adopts the distance between household residence and the village committee as the instrumental variable. The selection of this instrument is based on two considerations: relevance—the distance affects policy outreach, regulatory frequency, and participation in collective activities, thereby systematically influencing both the intensity of environmental regulation and the degree of social network embeddedness; and exogeneity—the distance is primarily determined by pre-existing geographic and historical settlement patterns, and, after controlling for individual, household, and regional characteristics, it does not directly alter farmers’ ecological inputs or production technologies, nor does it affect ecological efficiency through channels other than environmental regulation or social network embeddedness. Table 6 reports the results of the endogeneity tests. The instrumental variable, distance between household residence and the village committee, is significantly and negatively correlated with both environmental regulation and social network embeddedness, and the first-stage F-statistics exceed 10, indicating strong instrument relevance. In the second stage, the fitted values of environmental regulation and social network embeddedness both show significant positive effects on ecological efficiency, confirming the robustness of the main findings. Overall, the results suggest that endogeneity does not substantially bias the estimated relationships.

**Table 6:** Results of endogeneity Tests.

Variable Name	Model (7)	Model (8)	Model (9)	Model (10)
	2SLS First Stage	2SLS Second Stage	2SLS First Stage	2SLS Second Stage
	Environmental Regulation	Ecological Efficiency	Social Network Embeddedness	Ecological Efficiency
Distance between Household Residence and Village Committee	–0.152*** (0.027)		-0.172*** (0.018)	
Fitted value of Environmental Regulation		0.196*** (0.049)		
Fitted value of Social Network Embeddedness				0.0627*** (0.011)
const			0.945 (0.765)	0.254*** (0.010)
First-stage F-statistic	13.42		19.28	
Second-stage Wald statistic		9.85		16.79
n	324	324	324	324
R^2^	0.245	0.045	0.357	0.152

Note: *, **, and *** indicate significance at the 10%, 5%, and 1% levels, respectively. The same notation applies hereafter.

### Robustness check

Given that the OLS regression assumes a continuous and uncensored dependent variable, while forest farmers’ eco-efficiency is inherently bounded, a Tobit regression model was employed to correct for potential estimation bias caused by censoring and to enhance robustness. The Tobit regression confirmed that both environmental regulation and social network embeddedness exert significant positive effects on eco-efficiency. These findings align with the baseline OLS results, indicating that the direction and significance of the main variables remain stable across model specifications and confirming the reliability of the estimated outcomes (Table 7).

**Table 7:** Tobit regression results.

Variable Name	Model (11)	Model (12)	Model (13)	Model (14)
	Forest Farmer Eco-efficiency			
Environmental Regulation	0.021***(0.003)			
Coercive Regulation		0.022***(0.015)		
Incentive-based Regulation		0.046***(0.013)		
Guidance-based Regulation		0.052***(0.009)		
Social Network Embeddedness			0.023***(0.007)	
Degree Centrality				1.085***(0.290)
Betweenness Centrality				0.350**(0.200)
Closeness Centrality				0.026***(0.193)
Control Variables	Control variables included in the model	Control variables included in the model	Control variables included in the model	Control variables included in the model
Constant	-0.016(0.019)	0.011(0.010)	0.122(0.010)	-0.002(0.047)
n	324	324	324	324
R^2^	0.297	0.335	0.091	0.396

### Interaction effects analysis

Given the potential interaction between environmental regulation and social network embeddedness in influencing ecological efficiency among forest farmers, interaction terms were constructed to empirically test Hypothesis H3. Table 8 presents regression results for the interaction terms between degree centrality, betweenness centrality, and closeness centrality with the three types of environmental regulation: coercive, incentive-based, and guidance-based. The objective is to examine the moderating roles and heterogeneity of different network structure characteristics in shaping the effectiveness of various regulatory approaches.

**Table 8:** Stepwise regression results.

Variable Name	Model (15)		Model (16)	
	Forest Farmer Eco-efficiency			
	Coefficient	Std. Error	Coefficient	Std. Error
Coercive environmental regulation× Degree Centrality	0.068***	0.011	0.069***	0.011
Coercive environmental regulation× Betweenness Centrality	0.005	0.022	0.007	0.021
Coercive environmental regulation× Closeness Centrality	0.048	0.026	0.043	0.020
Incentive-based Regulation × Degree Centrality	0.058***	0.010	0.059***	0.010
Incentive-based Regulation× Betweenness Centrality	0.032***	0.008	0.033***	0.009
Incentive-based Regulation × Closeness Centrality	0.016	0.011	0.016	0.011
Guidance-based Regulation × Degree Centrality	0.036***	0.008	0.037***	0.008
Guidance-based Regulation × Betweenness Centrality	0.049***	0.009	0.052***	0.009
Guidance-based Regulation × Closeness Centrality	0.041***	0.008	0.040***	0.008
Control Variables	Control variables included in the model		Control variables included in the model	
Sample Size	324		324	

The regression results indicated a significantly positive interaction between coercive environmental regulation and degree centrality. Farmers with a higher degree of centrality, due to frequent information exposure and heightened public visibility, gained timely access to regulatory information while facing stronger community supervision and peer pressure. Under the combined influence of governmental enforcement and social monitoring, their compliance intentions were reinforced, thereby enhancing ecological efficiency. In contrast, the interaction terms between coercive regulation and both betweenness centrality and closeness centrality were statistically insignificant. One possible explanation lies in the limited reliance of coercive measures on information diffusion, even when individuals occupy key network positions. Although closeness centrality improves access to policy-related information, the rigid nature of coercive regulation may restrict the translation of such informational advantages into practical behavioral changes.

Incentive-based regulation exhibited significantly positive interaction effects with both degree centrality and betweenness centrality. Economic and reputational incentives boosted farmers’ willingness to adopt environmentally sustainable practices. Individuals with broader social ties accessed policy updates more efficiently and responded quickly, converting incentives into ecological gains. Those situated at critical points in information networks effectively integrated resources and mitigated risks, thereby enhancing their capacity to capitalize on available policy support. However, the interaction between incentive-based regulation and closeness centrality was not significant. While closeness centrality facilitates faster information access, the effectiveness of incentive mechanisms depends more on the depth of participation and accessibility of resources. In cases where incentives are unevenly distributed, or access thresholds are high, information advantages alone may not translate into improved ecological outcomes.

All interaction terms between guidance-based regulation and the three centrality measures showed significantly positive effects. Guidance-oriented regulation relies on information dissemination, awareness campaigns, and technical training to encourage sustainable behavior, emphasizing behavioral transformation through knowledge and perception enhancement. Degree centrality facilitated the timely receipt of training and technical support from governmental or cooperative bodies, accelerating knowledge acquisition and behavioral adaptation. Betweenness and closeness centrality further improved information integration and transmission efficiency, reinforcing responsiveness to regulatory guidance. Higher centrality in any dimension corresponded with increased responsiveness and engagement, leading to notable improvements in ecological efficiency under guidance-based regulatory regimes.

## Discussion

The present study empirically examined the mechanisms through which social network embeddedness influences the ecological efficiency of hazelnut production among forest farmers. Compared with previous literature, the research introduces several methodological and theoretical innovations.

(1) Previous analyses frequently relied on subjective questionnaire-based assessments to evaluate social network embeddedness; a method vulnerable to personal bias^31–33^. The current research applied a social network analysis approach, using degree centrality, betweenness centrality, and closeness centrality to quantify farmers’ network positions objectively. This approach improved the scientific rigor of the data analysis and strengthened the reliability of the results.

(2) The empirical analysis demonstrated that coercive, incentive-based, and guidance-based environmental regulations each exerted a significant positive effect on ecological efficiency, consistent with previous findings^39,40,43^. Among them, coercive regulation showed the most substantial influence, suggesting that rigid standards and mandatory enforcement continue to serve as key drivers during the current phase of ecological transition.

(3) This study proposes an integrated analytical framework that links external regulatory pressure, internal social network embeddedness, and ecological efficiency. The analysis demonstrates that farmers’ network positions significantly moderate the effectiveness of regulatory policies, highlighting the need for localized and targeted interventions. By moving beyond the oversimplified efficiency pathways emphasized in prior studies^27,34^, the framework not only strengthens the practical relevance of the findings but also deepens theoretical understanding: it extends environmental governance research by showing how different types of regulatory instruments jointly shape ecological outcomes, while simultaneously enriching social capital theory by revealing how farmers’ network embeddedness conditions the translation of external policies into tangible efficiency gains.

(4) Despite its contributions, the study has two main limitations. First, the long production cycle in forestry poses challenges for cross-sectional data to capture the lag between input and output, which restricts the ability to reflect long-term changes in ecological efficiency. Future research could adopt dynamic panel data models to examine long-term effects and mechanisms. Second, while social network analysis enabled the objective measurement of network positions, the study did not fully capture aspects of relationship quality, such as trust intensity, reciprocity, and network evolution. Future work may benefit from integrating relational embeddedness and dynamic network perspectives to refine the measurement framework.

## Conclusion and policy implications

Based on survey data from 324 hazelnut farmers in Tieling City, Liaoning Province, the study employed a super-efficiency SBM model to measure ecological efficiency and systematically examined the effects of environmental regulation and social network embeddedness. The main conclusions are as follows:

(1) Environmental regulation significantly improved ecological efficiency, with effects varying across regulatory types. Coercive regulation, which relies on mandatory policies and punitive measures, has proven most effective in promoting compliance with environmental standards. Incentive-based regulation, through subsidies and rewards, reduced the costs of green transition and enhanced farmers’ willingness to adopt sustainable practices. Guidance-based regulation improved environmental awareness and cognitive capacity through information campaigns and technical training, thereby supporting ecological performance.

(2) Social network embeddedness positively influenced ecological efficiency. All three centrality indicators (degree, betweenness, and closeness centrality) had significant positive effects. Higher degree of centrality expanded social connections, facilitating access to policy, technical, and market resources. Betweenness centrality enhanced the circulation of information and resources, strengthening farmers’ ability to obtain external support and foster cooperation. Closeness centrality improved the timeliness of information response, enabling the effective application of ecological technologies and promoting continuous improvements in ecological efficiency.

(3) The interaction between environmental regulation and social network embeddedness significantly affected ecological efficiency. Under coercive regulation, the interaction with degree centrality showed a significant positive effect. Farmers in more central network positions were better able to access policy information and were subject to stronger social pressure, increasing their likelihood of compliance and ecological gains. Interaction effects with betweenness and closeness centrality were not significant. This may be explained by the rigid nature of coercive regulation, which relies more on uniform enforcement and administrative penalties than on information diffusion. As a result, even if farmers occupy advantageous network positions, their informational benefits are less likely to translate into differentiated ecological outcomes under coercive rules. Under incentive-based regulation, degree and betweenness centrality interacted positively and significantly with ecological efficiency. Farmers with strong capacities for information acquisition and resource integration were more responsive to incentives and better able to translate them into ecological gains. The interaction with closeness centrality was not significant, possibly because the impact of incentive policies depends more on participation intensity and resource access. When resource allocation is uneven, or entry thresholds are high, the advantage of faster information access alone may not lead to effective outcomes. All interaction terms between guidance-based regulation and the three centrality measures were significantly positive. This regulatory approach relies on information dissemination, technical guidance, and demonstration activities to encourage behavioral change. Farmers occupying central positions in social networks benefited from greater exposure to policy signals and were more likely to internalize and act upon them, thereby achieving notable improvements in ecological efficiency.

Based on these findings, the following policy recommendations are proposed: First, the enforcement of environmental regulation should be strengthened. Clear environmental standards and tiered penalty mechanisms should be established to ensure compliance and enhance the effectiveness of coercive measures. Concurrently, long-term incentive frameworks should be constructed through increased fiscal support, green technology dissemination, and low-interest credit to reduce the costs of green production and support sustained transformation. Guidance-based regulation should focus on expanding public education, technical training, and demonstration programs to enhance farmers’ environmental awareness and knowledge. Second, the construction of social networks should be reinforced to enhance farmers’ network embeddedness. Support for farmer communication activities, cooperative platforms, and demonstration workshops should be expanded to improve connectivity, accelerate information flow, and deepen both the breadth and depth of social networks. Third, regulatory strategies should be differentiated in accordance with the interaction effects between environmental regulation and social networks. For coercive regulation, enforcement mechanisms should be reinforced, and farmers with a high degree of centrality should be engaged as role models to utilize their information advantages and network influence in promoting broader compliance. For incentive-based regulation, resources should be allocated more efficiently, with priority given to farmers who demonstrate stronger capacities for resource integration and greater potential for demonstration within their networks, while policy entry thresholds should be lowered to enable more farmers to translate incentives into tangible ecological gains. For guidance-based regulation, broad-based education and awareness-building efforts should be emphasized, and farmers occupying central positions in social networks should be prioritized as key recipients of training and demonstration activities, thereby leveraging their information diffusion effects to stimulate wider behavioral change. A comprehensive, accessible, and diversified training system should be established to enhance farmers’ understanding of environmental policies, green technologies, and sustainability principles, thus laying a cognitive and technical foundation at the community level to support long-term improvements in ecological efficiency.

## Data Availability

The data used in this study were obtained through a field survey conducted in January 2024 among hazelnut farmers in Tieling City, Liaoning Province. Due to privacy concerns and institutional data management policies, the dataset is not publicly available and cannot be shared by the authors. Researchers interested in the data may contact the corresponding institution for potential access under specific agreements, subject to approval.
